# *Figla* promotes secondary follicle growth in mature mice

**DOI:** 10.1038/s41598-021-89052-3

**Published:** 2021-05-10

**Authors:** Asuka Okunomiya, Akihito Horie, Hirohiko Tani, Yukiyasu Sato, Shiro Takamatsu, J. B. Brown, Miki Sugimoto, Junzo Hamanishi, Eiji Kondoh, Noriomi Matsumura, Masaki Mandai

**Affiliations:** 1 Department of Gynecology and Obstetrics, Kyoto University Graduate School of Medicine, 54 Shogoin Kawahara-cho, Sakyo, Kyoto, 606-8507 Japan; 2 Life Science Informatics Research Unit, Department of Molecular Biosciences, Kyoto University Graduate School of Medicine, Kyoto, Japan; 3 Laboratory of Animal Physiology and Functional Anatomy, Kyoto University Graduate School of Agriculture, Kyoto, Japan; 4 Department of Obstetrics and Gynecology, Kindai University Faculty of Medicine, Osaka, Japan

**Keywords:** Developmental biology, Oogenesis, Computational biology and bioinformatics, Transcriptomics

## Abstract

The in vitro growth (IVG) of human follicles is a potential fertility option for women for whom cryopreserved ovarian tissues cannot be transplanted due to the risk of cancer cell reintroduction; however, there is currently no established method. Furthermore, optimal IVG conditions may differ between the follicles of adult and pre-pubertal females due to molecular differences suggested by basic research. To systematically identify differences between the secondary follicles of adult and pre-pubertal females, a comparative transcriptomic study using mice was conducted herein. Among differentially expressed genes (DEGs), *Figla* was up-regulated in mature mice. We successfully down-regulated *Figla* expression in secondary follicle oocytes by a *Figla* siRNA microinjection, and the subsequent IVG of follicles showed that the diameter of these follicles was smaller than those of controls in mature mice, whereas no significant difference was observed in premature mice. The canonical pathways of DEGs between control and *Figla*-reduced secondary follicles suggest that *Figla* up-regulates VDR/RXR activation and down-regulates stem cell pluripotency as well as estrogen signaling. We demonstrated for the first time that folliculogenesis of the secondary follicles of premature and mature mice may be regulated by different factors, such as *Figla* with its possible target genes, providing insights into optimal IVG conditions for adult and pre-pubertal females, respectively.

## Introduction

An in vitro culture of human follicles may be a viable option for women for whom cryopreserved ovarian tissues cannot be transplanted due to the risk of reintroducing cancer cells^1^; however, there is currently no definitively developed and established method^2^. In 2014, Anderson et al.^3^ was the first to compare the in vitro growth potential of follicles from pre-pubertal girls with that of adults by culturing ovarian biopsies for 6 days, with secondary follicles then being isolated and cultured for a further 6 days. The findings obtained showed that follicles from women of all ages were induced to grow in vitro; however, the growth potential of pre-pubertal-derived follicles was significantly weaker than that of adult-derived follicles, indicating that follicle growth-regulating genes in pre-pubertal girls differ from those in adults, and, thus, optimal culture conditions may also differ. In mice, a previous study reported that responsiveness to growth factors, such as activin A and TGF-β, under in vitro culture conditions differed between the secondary follicles of mature and premature mice^4^. As the hormonal environment of follicles in the ovary differs between pre-pubertal and adult females^5^ because the ovary is placed in the hypothalamus-pituitary-gonad axis after the onset of puberty in mammals^6, 7^, follicle growth-regulating genes in pre-pubertal and adult females may likely differ. A comparison of gene expression profiles between adult- and pre-pubertal-derived secondary follicles will facilitate the development of efficient in vitro follicle culture systems^8, 9^ that are optimal for adult and pre-pubertal females. Among mammals, the gene expression of follicles at each follicle developmental stage, from primordial to late antral follicles, has been reported in mice^10^ and humans^11^, and changes in gene expression during follicle development have been presented as a roadmap; however, a comparison of matching developmental stage follicles (e.g., secondary follicles) between mature and premature females has not yet been conducted. Therefore, based on cross-species comparative follicle dynamics^4, 12^, we herein conducted a comparative transcriptomic analysis of secondary follicles between mature and premature mice followed by in vitro follicle cultures in an attempt to identify follicle growth-regulating genes that function differently between mature and premature mice. The results obtained may promote the development of efficient in vitro follicle culture systems that are optimal for adult and pre-pubertal females.

## Results

### Comparative transcriptomic analysis of secondary follicle oocytes between mature and premature mice

**Figure 1:**
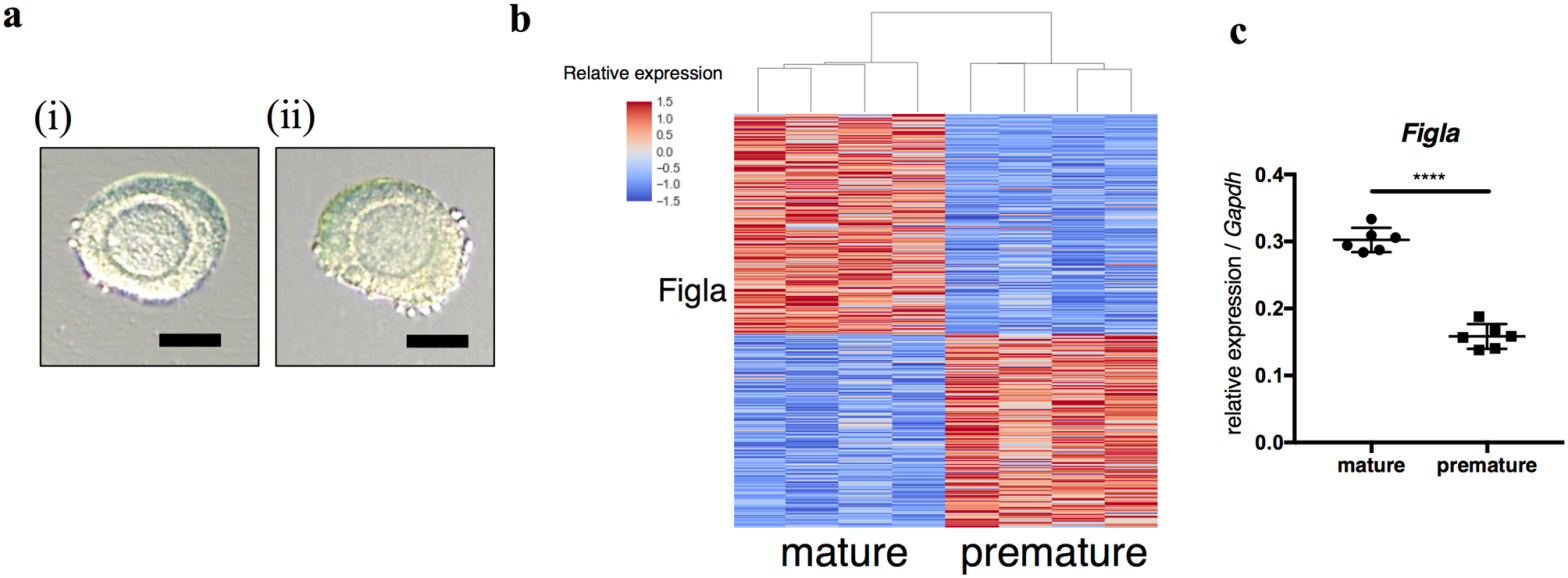
Differentially expressed genes in secondary follicle oocytes between mature and premature mice. (**a**) Representative pictures of the secondary follicles of (i) a mature mouse and (ii) premature mouse. Scale bars, 50 µm. (**b**) Heatmap of differentially expressed genes (DEGs, log2 fold change > 0.5, false discovery rate (FDR) < 0.01). Four samples from mature and premature mice were compared. (**c**) Expression levels of *Figla* in the secondary follicle oocytes of mature mice and premature mice by qRT-PCR (n = 6, in each group).

Morphologically equivalent normal secondary follicles were collected from mature and premature mice (Fig. 1a). The gene expression profiles of secondary follicle oocytes were compared between mature and premature mice by RNA sequencing. Follicles collected from one mouse comprised one sample and four samples were compared. Using DESeq2 (PMID: 25516281), defining differentially expressed genes (DEGs) as those with FPKM > 1 in at least one sample, log2 fold change > 0.5, and false discovery rate (FDR) < 0.01, there were 1368 DEGs, of which 728 and 640 were up-regulated in mature and premature mice, respectively (Fig. 1b, S1). Among them, we identified a germ cell-specific transcription factor with the gene ontology term “oogenesis”, which was *Figla* (Fig. 1c). The top 15 canonical pathways of DEGs by an Ingenuity Pathway Analysis (IPA) showed that the oxidative stress response and PI3K signaling were up-regulated in mature mice (Table 1, positive z-score), while RhoGDI signaling was up-regulated in premature mice (Table 1, negative z-score). A gene ontology analysis revealed that glutathione and lipid metabolic processes were up-regulated in mature mice (Table S1-1), while cAMP metabolic process was up-regulated in premature mice (Table S1-2).

**Table 1:** Top 15 canonical pathways of differentially expressed genes between secondary follicle oocytes of mature and premature mice.

Ingenuity canonical pathways	*p* value	Ratio^a^	z-score^b^	Genes^c^
Signaling by Rho family GTPases	1.05E−07	0.139	1.8	Acta2, **Arhgef17**, Arhgef4, **Arhgef5**, **Baiap2**, **Cdc42ep1**, Cdh8, **Ezr**, **Fnbp1**, **Fos**, Gnat2, Gnb1l, **Gnb5**, Gng3, Itga5, **Jun**, **Map3k20**, **Map3k21**, **Mras**, **Nfkb2**, **Pak3**, **Pi4ka**, Pip5k1b, **Pkn1**, Prkci, **Prkcz**, **Rac3**, Rhobtb1, **Rnd1**, **Rnd2**, Septin12, Septin2
IL-8 signaling	3.80E−07	0.145	1.964	**Braf**, **Ccnd2**, **Ccnd3**, **Cstb**, Egf, **Fnbp1**, **Fos**, Gnb1l, **Gnb5**, Gng3, **Gpld1**, Hmox1, **Itgav**, **Jun**, **Lasp1**, Mpo, **Mras**, Pld2, Prkci, **Prkcz**, **Rac3**, **Rasd1**, **Rasd2**, Rhobtb1, **Rnd1**, **Rnd2**, Tek, **Vasp**, **Vegfa**
Tec kinase signaling	3.16E−06	0.146	0.243	Acta2, Btk, **Fnbp1**, **Fos**, Frk, Gnat2, Gnb1l, Gnb5, Gng3, **Gtf2i**, Itga5, **Mras**, **Nfkb2**, **Pak3**, Prkci, **Prkcz**, **Rac3**, Rhobtb1, **Rnd1**, **Rnd2**, **Stat3**, Tnfrsf21, Vav3
Gαq signaling	5.50E−06	0.146	0.775	**Adra1b**, Btk, **Fnbp1**, Gnb1l, **Gnb5**, Gng3, **Gpld1**, **Grk2**, Hmox1, **Mras**, **Nfatc2**, **Nfkb2**, **Nfkbia**, Nfkbid, Plcb1, Pld2, **Ppp3ca**, Prkci, **Prkcz**, **Rac3**, Rhobtb1, **Rnd1**, **Rnd2**
NRF2-mediated oxidative stress response	1.23E−05	0.132	1.291	Acta2, **Cat**, **Cbr1**, **Dnajb1**, **Dnajb14**, **Dnajb2**, **Erp29**, **Fos**, **Ftl**, **Gsr**, **Gstm1**, **Gsto1**, **Herpud1**, Hmox1, **Jun**, **Junb**, **Keap1**, **Mras**, **Nqo2**, Prdx1, Prkci, **Prkcz**, **Rasd1**, **Rasd2**
PI3K signaling in B Lymphocytes	2.34E−05	0.145	1	Btk, **Card10**, **Cd81**, **Fos**, Inpp5d, **Jun**, **Mras**, **Nfatc2**, **Nfkb2**, **Nfkbia**, Nfkbid, Plcb1, Plce1, Plekha2, **Ppp3ca**, Prkci, **Prkcz**, **Rasd1**, **Rasd2**, Vav3
Relaxin signaling	2.57E−05	0.14	2.309	**Braf**, **Fos**, Gnat2, Gnb1l, **Gnb5**, Gng3, **Gucy1a1**, Gucy2c, **Jun**, **Mras**, **Nfkb2**, **Nfkbia**, Nfkbid, **Nos2**, Pde10a, Pde1a, Pde1b, Pde1c, **Prkcz**, **Smarcc2**, **Vegfa**
TNFR1 signaling	3.47E−05	0.22	1.508	Casp8, **Fos**, **Jun**, Naip1, **Nfkb2**, **Nfkbia**, Nfkbid, **Pak3**, **Ripk1**, **Tradd**
Pyrimidine ribonucleotides de novo biosynthesis	5.75E−05	0.227	1.265	**Ak7**, **Cad**, Cmpk2, Ctps2, Entpd1, **Nme2**, **Nme3**, **Nme4**, **Slc25a42**, **Smarca1**
RhoGDI signaling	0.000132	0.122	− 0.775	Acta2, **Arhgef17**, Arhgef4, **Arhgef5**, Cdh8, **Ezr**, **Fnbp1**, Gnat2, Gnb1l, **Gnb5**, Gng3, Itga5, **Mras**, **Pak3**, **Pi4ka**, Pip5k1b, **Rac3**, Rhobtb1, **Rnd1**, **Rnd2**
Axonal guidance signaling	0.000158	0.0909	N/A	Adam22, Adamts3, **Baiap2**, Bmp5, **Bmp7**, **Efna1**, **Efnb2**, Egf, Epha10, Epha4, **Fzd5**, Gnat2, Gnb1l, **Gnb5**, Gng3, Itga5, **Mmp17**, **Mras**, **Nfatc2**, Nrp1, **Pak3**, **Pdgfa**, **Pfn2**, Plcb1, Plce1, **Plxnc1**, **Ppp3ca**, Prkci, **Prkcz**, **Rac3**, **Rasd1**, **Rasd2**, **Rnd1**, **Rtn4r**, Sema3a, **Sema3b**, **Sema4c**, Sema6a, Stk36, **Tubb2a**, **Vasp**, **Vegfa**
Pyrimidine ribonucleotides interconversion	0.000219	0.214	1	**Ak7**, Cmpk2, Ctps2, Entpd1, **Nme2**, **Nme3**, **Nme4**, **Slc25a42**, **Smarca1**
Molecular mechanisms of cancer	0.000240	0.0946	N/A	**Arhgef17**, Arhgef4, **Arhgef5**, Bmp5, **Bmp7**, **Braf**, Casp8, **Ccnd2**, **Ccnd3**, Cdk15, **Ctnna2**, **Fnbp1**, **Fos**, **Fzd5**, **Gab1**, **Gab2**, Gnat2, Itga5, **Jun**, Lef1, **Mras**, Naip, **Nfkb2**, **Nfkbia**, Nfkbid, **Pak3**, Plcb1, Prkci, **Prkcz**, **Rac3**, **Rasd1**, **Rasd2**, Rhobtb1, **Rnd1**, **Rnd2**, Stk36
Endothelin-1 signaling	0.000251	0.117	0.447	**Abhd3**, **Braf**, Casp8, **Fos**, **Gab1**, Gnat2, **Gpld1**, **Gucy1a1**, Gucy2c, Hmox1, **Jun**, **Mras**, **Nos1**, **Nos2**, Plcb1, Plce1, Pld2, Prkci, **Prkcz**, **Rasd1**, **Rasd2**, Shc3
Regulation of actin-based motility by Rho	0.000288	0.149	1.155	Acta2, **Baiap2**, **Fnbp1**, Itga5, **Pak3**, **Pfn2**, **Pi4ka**, Pip5k1b, **Rac3**, Rhobtb1, **Rnd1**, **Rnd2**

*N/A* not available.

^a^Ratio of listed genes found in each pathway over the total number of genes in that pathway.

^b^z-score positive when pathways are up-regulated in mature mice and negative when pathways are up-regulated in premature mice.

^c^Genes in bold are up-regulated in mature mice, while genes in normal font are up-regulated in premature mice.

### In vitro follicle culture and siRNA microinjection

**Figure 2:**
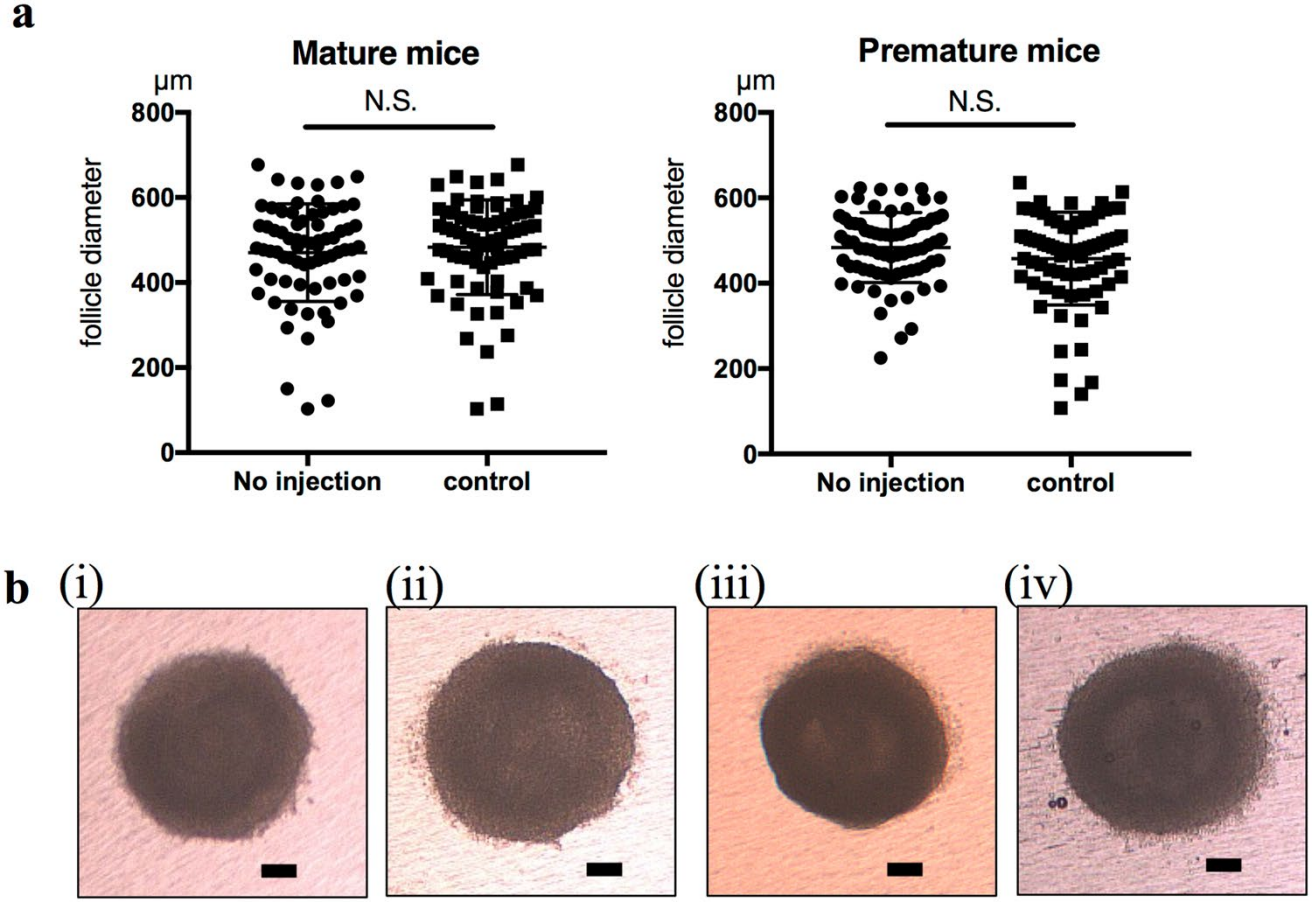
Follicle growth in no injection and control groups. (**a**) The diameter of cultured follicles did not significantly differ between follicles in the no injection group and negative control siRNA-injected follicles in both mature (left) and premature (right) mice. The diameters of mature mice follicles were 470.4 ± 114.8 µm (n = 73) in the no injection group and 482.8 ± 111.3 µm (n = 74) in the control group. The diameters of premature mice follicles were 483.8 ± 81.99 µm (n = 75) in the no injection group and 457.5 ± 108.7 µm (n = 76) in the control group. (**b**) Representative pictures of follicles after 12 days of in vitro culture. (i) Mature mouse follicle of the no injection group, (ii) mature mouse follicle of the control group, (iii) premature mouse follicle of the no injection group, and (iv) premature mouse follicle of the control group. Scale bars, 100µm.

When secondary follicles were cultured for 12 days using the in vitro culture method selected in the present study, no significant differences were observed in the diameter of follicles between mature and premature mice (Figure S2). Negative control siRNA (siRNA with no silencing effects) was microinjected into the oocytes of secondary follicles with subsequent culturing. No significant differences were observed in follicle diameters between no injection follicles and control follicles after 12 days of culture in both mature and premature mice (Fig. 2).

### In vitro culture of si-*Figla* follicles

**Figure 3:**
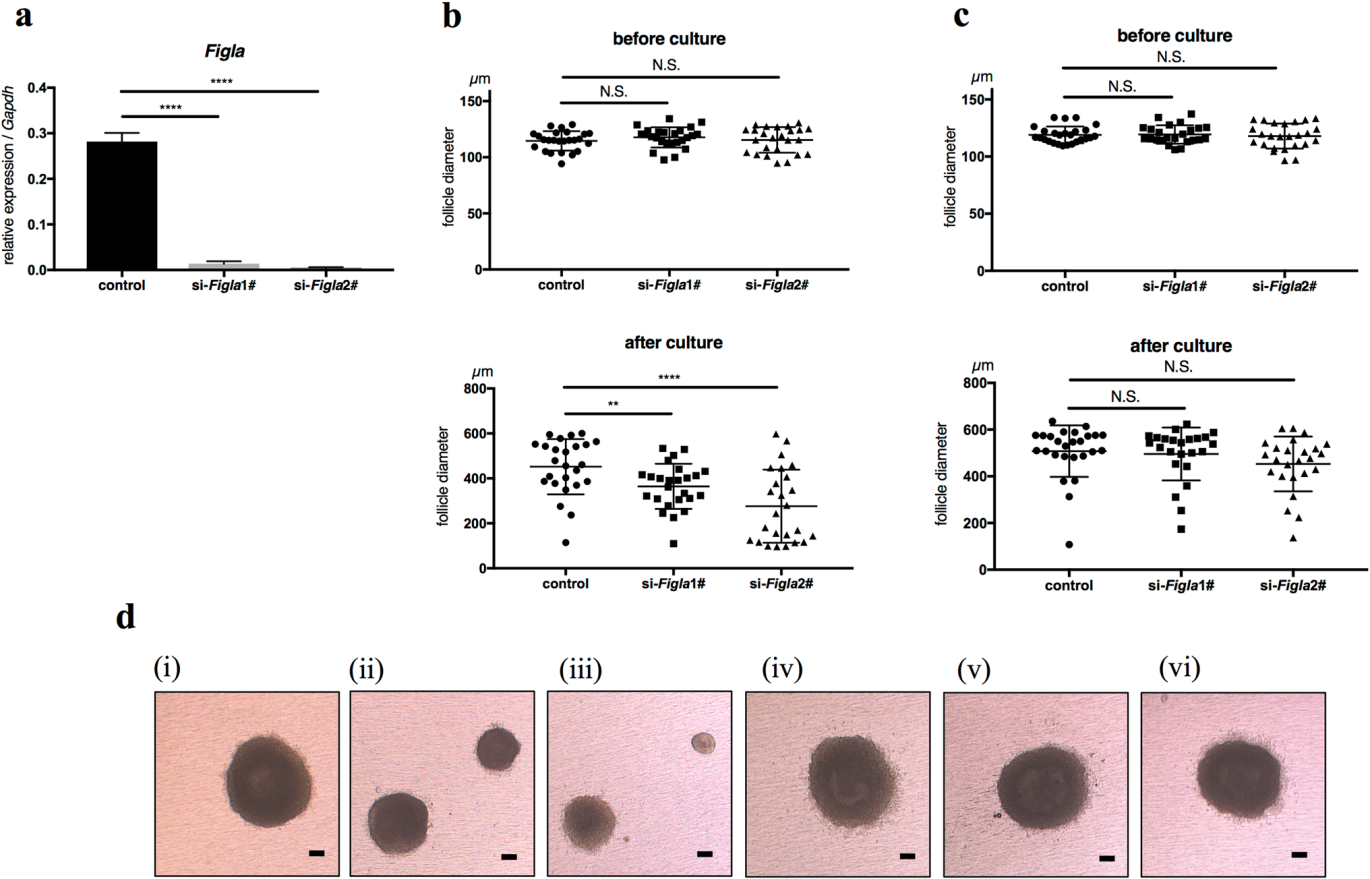
Reduced follicle growth of si-*Figla* in mature mice. (**a**) The expression of *Figla* was significantly weaker in si-*Figla* (1# left, 2# right) secondary follicle oocytes than in the control (n = 3, for each group). (**b**, **c**) The diameters of follicles before culture (upper graph) and after 12 days of in vitro culture (lower graph) are shown. The diameter of si-*Figla* was significantly smaller than the control after culture in mature mice (**b**), whereas no significant difference was observed in premature mice (**c**). The diameters of mature mouse follicles before culture were 114.6 ± 8.69 µm (n = 25) in control, 117.8 ± 8.98 µm (n = 25) in si*Figla*1#, and 115.4 ± 11.35 µm (n = 25) in si-*Figla*2#, whereas diameters after culture were 452.1 ± 123.2 µm (n = 25) in control, 364.7 ± 100.4 µm (n = 25) in si-*Figla*1#, and 276.2 ± 162.8 (n = 25) µm in si-*Figla*2#. The diameters of premature mice follicles before culture were 119.0 ± 7.31 µm (n = 26) in control, 119.4 ± 8.07 µm (n = 24) in si-*Figla*1#, and 117.9 ± 11.0 µm (n = 25) in si-*Figla*2#, whereas diameters after culture were 508.0 ± 110.5 µm (n = 26) in control, 495.8 ± 113.2 µm (n = 24) in si-*Figla*1#, and 453.2 ± 117.4 µm (n = 25) in si-*Figla*2#. (**d**) Representative pictures of follicles after 12 days of in vitro culture, (i)–(iii) mature mice, (iv)–(vi) premature mice, (i), (iv) control, (ii), (v) si-*Figla*1#, (iii), (vi) si-*Figla*2#. Scale bars, 100 µm.

Two *Figla* siRNAs (1# and 2#) were prepared and microinjected into the oocytes of secondary follicles. After an incubation, oocytes of both si-*Figla*1# and si-*Figla*2# follicles showed the significantly weaker expression of *Figla* than control follicles (Fig. 3a). Three groups of secondary follicles, which were si-*Figla*1#, si-*Figla*2#, and control, were prepared for mature and premature mice, respectively. No significant differences were noted in the diameters of secondary follicles before culture between the groups for mature and premature mice (Fig. 3b,c). After 12 days of in vitro culture, the follicle diameters of si-*Figla*1# (364.7 ± 100.4 µm, n = 25) and si-*Figla*2# (276.2 ± 162.8 µm, n = 25) in mature mice were significantly smaller than that in the control (452.1 ± 123.2 µm, n = 25) (Fig. 3b,d). In contrast, the follicle diameters of si-*Figla*1# (495.8 ± 113.2 µm, n = 24) and si-*Figla*2# (453.2 ± 117.4 µm, n = 25) in premature mice were not significantly different from that in the control (508.0 ± 110.5 µm, n = 26) (Fig. 3c,d). Three independent experiments were performed with similar results.

### Downstream genes of *Figla* in secondary follicles and neonatal ovaries

**Figure 4:**
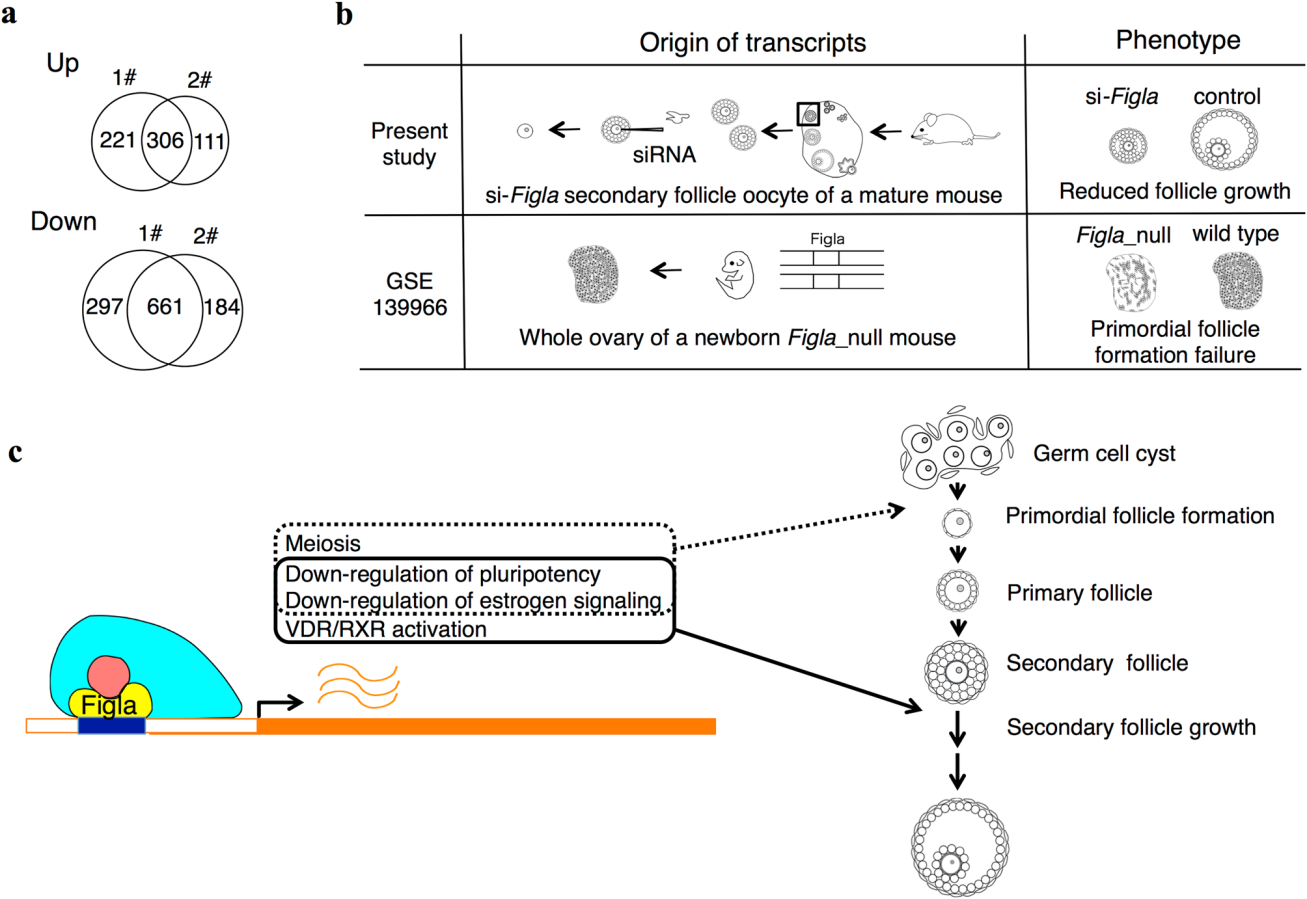
Pursuit of downstream genes of *Figla*. (**a**) Venn diagram of DEGs in two comparisons, si-*Figla*1# vs control (1#) and si-*Figla*2# vs control (2#). Four samples for each were compared. In total, 306 genes were up-regulated and 661 genes were down-regulated in these two si-*Figla*s. (**b**) Schematic of transcripts and phenotypes of the present study and GSE 139966. (**c**) Schematic of the hypothetical model of *Figla* functions in folliculogenesis. *Figla* may regulate the follicle growth of secondary follicles by down-regulating pluripotency and estrogen signaling and up-regulating VDR/RXR activation (solid line), whereas *Figla* may regulate primordial follicle formation through the activation of meiosis and down-regulation of pluripotency and estrogen signaling (dotted line).

In order to investigate the downstream genes of *Figla* in secondary follicles, secondary follicles collected from four mice were combined and randomly distributed to si-*Figla*1#, si-*Figla*2#, and control. After a 48-hr incubation, follicles were denuded to obtain oocytes for a gene expression analysis. Four samples for each were prepared. Gene expression profiles were compared between si-*Figla*1# and control and between si-*Figla*2# and control using DESeq2. Based on the definition of DEGs as those with FPKM > 1 for at least one sample and FDR < 0.1, 306 genes were up-regulated and 661 were down-regulated in these two si-*Figla*s (Fig. 4a). A pathway analysis by IPA was performed using these DEGs (Table 2). A positive z-score indicates pathways down-regulated in si-*Figla* = pathways expected to be up-regulated by *Figla*, whereas a negative z-score indicates pathways up-regulated in si-*Figla* = pathways expected to be down-regulated by *Figla*. In the top 15 canonical pathways, stem cell signaling (Mouse Embryonic Stem Cell Pluripotency and Wnt/β-catenin Signaling^13, 14^) and estrogen receptor signaling were expected to be down-regulated by *Figla*, while VDR/RXR activation was expected to be up-regulated.

**Table 2:** Top 15 canonical pathways of differentially expressed genes between secondary follicles with *Figla* suppression and control.

Ingenuity canonical pathways	*p* value	Ratio^a^	z-score^b^	Genes^c^
Molecular mechanisms of cancer	1.32E−09	0.11	N/A	Abl1, Adcy6, Apc, **Aph1c**, Arhgef11, Arhgef18, Atm, Atr, **Birc3**, Brca1, Cbl, **Ccne2**, Cdc25b, Cdk12, **Cdk5**, **Cdkn2b**, Crebbp, Ctnnd1, Ep300, **Fzd5**, Fzd7, Lrp1, Lrp5, Lrp6, **Mapk11**, Pak2, Pik3c2a, Prkd3, Prkdc, Ptch1, **Raf1**, **Rhobtb1**, **Rhoh**, **Rras2**, Smad3, Smad5, Sos1, Tcf4, **Tgfb2**, **Wnt7a**, **Wnt8a**, Xiap, **Zbtb17**
Actin cytoskeleton signaling	1.78E−05	0.106	− 1.414	Actr2, Apc, Arhgap24, **Bcar1**, Diaph3, **Fgf8**, Flna, Iqgap3, Mprip, Myh11, Myh9, Pak2, **Pdgfb**, Pik3c2a, **Raf1**, **Rras2**, Sos1, Ssh2, Tln1, Tln2, Trio, Ttn, Vav2
Estrogen receptor signaling	4.47E−05	0.0884	− 3.528	Adcy6, Cacna1d, Crebbp, **Eif2b4**, Ep300, Igf1r, Igf2r, Lepr, Med12, Med12l, Med13, Med13l, Med14, Mprip, Mtor, Ncoa1, Ncoa2, Ncoa3, Ncor2, Nr3c1, Pelp1, Pik3c2a, Prkd3, Prkdc, **Raf1**, **Rras2**, Sos1, Tbl1xr1, Thrap3
Mouse embryonic stem cell pluripotency	5.13E−05	0.136	− 1.387	Apc, Crebbp, **Fzd5**, Fzd7, **Id2**, Il6st, **Mapk11**, Pik3c2a, **Raf1**, **Rras2**, Smad5, Sos1, Tcf4, Xiap
Wnt/β-catenin signaling	5.37E−05	0.11	− 2.357	Apc, Appl1, Bcl9, Crebbp, Csnk2a1, Ep300, **Fzd5**, Fzd7, Kremen1, Lrp1, Lrp5, Lrp6, Nr5a2, **Ppp2r1b**, **Sox15**, Tcf4, **Tgfb2**, **Wnt7a**, **Wnt8a**
Cell cycle: G2/M DNA damage checkpoint regulation	0.000107	0.184	− 0.707	Abl1, Atm, Atr, Brca1, Cdc25b, Ep300, **Plk1**, Prkdc, Trip12
NER Pathway	0.0002	0.126	− 1.732	**Cops4**, Cops8, Ddb1, Ep300, Ercc4, **Gps1**, Lig3, Pole, Polr2a, **Polr2d**, **Rfc4**, Rnf111, **Rpa1**
Glioblastoma multiforme signaling	0.000282	0.103	1	Apc, **Fzd5**, Fzd7, Igf1r, Itpr1, Mtor, **Pdgfb**, Pik3c2a, **Raf1**, **Rhobtb1**, **Rhoh**, **Rras2**, Sos1, Tsc1, Tsc2, **Wnt7a**, **Wnt8a**
Role of CHK proteins in cell cycle checkpoint control	0.000355	0.158	− 0.816	Atm, Atr, Brca1, Mdc1, **Plk1**, **Ppp2r1b**, **Rfc4**, **Rpa1**, Tlk1
Integrin signaling	0.000794	0.0892	− 1.213	Abl1, Actr2, **Arf1**, Arf3, **Bcar1**, Itgav, Mprip, Pak2, **Pdgfb**, Pik3c2a, Pikfyve, **Raf1**, **Rhobtb1**, **Rhoh**, **Rras2**, Sos1, Tln1, Tln2, Ttn
VDR/RXR activation	0.000933	0.128	1.414	Csnk2a1, Ep300, Lrp5, Ncoa1, Ncoa2, Ncoa3, Ncor2, Prkd3, Rxra, **Tgfb2**
RhoA signaling	0.00112	0.106	− 2.309	Abl2, Actr2, Arhgef11, Cit, Igf1r, Mprip, Pi4ka, Pikfyve, Pip4k2c, Rapgef2, Rapgef6, Septin12, Ttn
Glioma signaling	0.00129	0.109	− 1.265	Abl1, Camk1d, **Cdkn2b**, Igf1r, Igf2r, Mtor, **Pdgfb**, Pik3c2a, Prkd3, **Raf1**, **Rras2**, Sos1
TGF-β signaling	0.00138	0.115	− 0.333	Crebbp, Ep300, **Mapk11**, **Raf1**, Rnf111, **Rras2**, Smad3, Smad5, Sos1, **Tgfb2**, **Tgif1**
ATM signaling	0.00148	0.113	− 1.265	Abl1, Atm, Atr, Brca1, Crebbp, Herc2, **Mapk11**, Mdc1, **Ppp2r1b**, Tlk1

^a^Ratio of listed genes found in each pathway over the total number of genes in that pathway.

^b^z-score positive when pathways are down-regulated in the *Figla* siRNA-injected group (= pathways are expected to be up-regulated by *Figla*), while negative when pathways are up-regulated in the *Figla* siRNA-injected group (= pathways are expected to be down-regulated by *Figla*).

^c^Genes in bold are down-regulated in the *Figla* siRNA-injected group (= genes expected to be up-regulated by *Figla*), while genes in normal font are up-regulated in the *Figla* siRNA-injected group (= genes expected to be down-regulated by *Figla*).

To analyze downstream genes of *Figla* in neonatal ovaries (primordial follicle formation), we obtained RNA sequencing data from a previous study^15^ that compared gene expression in neonatal ovaries between *Figla*_null mice and controls (GSE 139966) (Fig. 4b). In a similar analysis in the present study, DEGs were obtained and a pathway analysis by IPA was performed (Table S2-1). A positive z-score indicates pathways down-regulated in *Figla*_null mice = pathways expected to be up-regulated by *Figla*, whereas a negative z-score indicates pathways up-regulated in *Figla*_null mice = pathways expected to be down-regulated by *Figla*. Stem cell signaling (Mouse Embryonic Stem Cell Pluripotency) was expected to be down-regulated by *Figla*. The GO analysis revealed that genes down-regulated in *Figla*_null mice (= genes expected to be up-regulated by *Figla*) included the DNA repair system and meiotic cell cycle (Table S2-2) as previously reported^15^, whereas genes up-regulated in *Figla*_null mice (= genes expected to be down-regulated by *Figla*) included responses to estrogen (Table S2-3).

Among *Figla* downstream genes, relative expression levels of *Gsr* and *Fancc* (genes related to oxidative stress response)*,* which were expected to be up-regulated by *Figla*, were significantly higher in mature mice than premature mice, whereas those of *Mmp2* and *Hmox1* (genes related to estrogen signaling) and *Rnf43* and *Bicc1* (genes related to stem cell signaling), which were expected to be down-regulated by *Figla*, were significantly lower in mature mice than premature mice (Figure S3).

## Discussion

Among the genes whose expression varied between the secondary follicle oocytes of mature and premature mice, *Figla*, a germ cell-specific transcription factor, was herein identified as a gene associated with oogenesis, suggesting a role in follicle growth, at least in the secondary follicles of mature mice. Furthermore, *Figla* may function differently in mature and premature mice according to differences in its expression level. A previous study reported the critical involvement of *Figla* in early oogenesis (primordial follicle formation), as shown by the loss of primordial follicles in the neonatal ovaries of *Figla*_null mice^16^. However, *Figla* was also found to be expressed in secondary follicles, which are in a later follicle developmental stage than primordial follicles, and in the ovaries of mature mice^17^. Although *Figla* appears to play an interesting role in secondary follicles, definitive evidence has not yet been obtained because of the difficulties associated with observing its function in *Figla*_null mice due to the disappearance of primordial follicles in the neonatal period^16^. In the present study, we examined the function of *Figla* in secondary follicles using a microinjection of *Figla* siRNA followed by an in vitro culture of injected follicles and a comparative transcriptomic analysis. The results obtained indicated that *Figla* was involved in follicle growth in mature mice, but exerted negligible effects on that in premature mice with its possible downstream target genes.

The mechanisms underlying the different effects of *Figla* between the secondary follicles of mature and premature mice have not yet been elucidated. This is mainly due to differences in its expression levels. In premature mice, its expression level may be under the threshold^18^ with a negligible contribution to follicle growth, which is compensated for by unknown regulating factors. RhoA signaling^19, 20^ (potentially down-regulated by *Figla*) with its adequate control by RhoGDI signaling^21^ (up-regulated in premature mice) may be one candidate. *Figla* may also contribute to the growth of the secondary follicles of premature mice when its expression is replenished by a microinjection of *Figla* mRNA^22^ or a plasmid^23^; however, technical difficulties have been associated with appropriately adjusting its intracellular distribution^15, 24^ and expression level. One possible explanation for the different expression levels of follicle growth-regulating genes such as *Figla* between pre-/mature mice is the presence of two distinct populations of primordial follicles^25, 26^ distributed in the medulla and cortex, respectively. The growing follicles (e.g., secondary and pre-antral follicles) present in premature mice are mainly supplied by the first population, whereas those in mature mice are mainly derived from the second population. These two distinct populations of primordial follicles may be conserved in mammals, including humans^12^, and, thus, the factors regulating folliculogenesis may also differ between the human follicles of pre-pubertal and adult females.

*Figla* has two different functions in two different settings; primordial follicle formation in neonatal ovary as previously described^15, 27^ and secondary follicle growth promotion in mature mice as shown in the present study. In order to investigate how *Figla* downstream genes overlap and differ in these two different settings, we both elucidated down stream genes of *Figla* in neonatal ovaries and secondary follicle oocytes of mature mice (Fig. 4b,c). Stem cell signaling (Mouse Embryonic Stem Cell Pluripotency and Wnt/β-catenin Signaling^13, 14^) was inhibited by *Figla* in both secondary follicles and neonatal ovaries, suggesting that *Figla* releases oocytes from an undifferentiated state, thereby promoting cell differentiation during early oogenesis and secondary follicle growth. Estrogen signaling was also down-regulated by *Figla* in secondary follicles and neonatal ovaries. A previous study reported that the ovaries of mature (9 weeks old) aromatase knockout mice showed follicles at each stage of development, from primordial to late antral follicles^28^. Therefore, estrogen is not essential for the formation of secondary follicles^29^, and it currently remains unclear whether it has any effects on secondary follicles. Additionally, since an estrogen stimulation during primordial follicle formation in the neonatal ovary was shown to impair follicle formation^30, 31^, an excessive estrogen stimulation in the secondary follicle may also impair follicle growth. These findings indicate that *Figla* promotes follicle formation by suppressing estrogen signaling in oocytes during early oogenesis, and also that the suppression of estrogen signaling in the secondary follicle may contribute to the appropriate regulation of follicle growth. VDR/RXR activation was up-regulated by *Figla* in secondary follicles, but not in neonatal ovaries. VDR is a nuclear receptor for vitamin D that promotes the transcription of its downstream target genes by forming heterodimers with RXR, the positive immunostaining of which has been reported in the secondary follicle oocytes of primates^32^. Studies on VDR knockout mice reported that primordial follicle formation during the neonatal state was not impaired, whereas follicles in developmental stages later than secondary follicles disappeared in the ovaries of 7-week-old mice^33^, suggesting that *Figla* advanced secondary follicles to later stages of development by VDR/RXR activation, a function that may be characteristic of secondary follicles. However, it currently remains unclear how this function is characteristic of secondary follicles. *Figla* is one of the basic helix-loop-helix (bHLH) transcription factors^17^. The binding motif of *Figla*, called E-box (CANNTG), is common to bHLH transcription factors^34^, and *Figla* functions with a cofactor called E12 when it regulates its downstream egg coat-coding genes, Zp1,2,3^16^. Similarly, VDR/RXR activation by *Figla* may be due to some cofactors or epigenetic modifications^35^ that are characteristic of secondary follicles. Meiotic cell cycle was up-regulated by *Figla* in neonatal ovaries but not in secondary follicles. This correlates to early oogenesis that germ cells enter meiosis and arrest at the diplotine stage of the first meiotic prophase when primordial follicles are created until the resumption of meiosis is induced in fully grown oocytes^36^.

Follicle growth dynamics has been studied using follicles obtained by juvenile individuals^37^ unless there were specific reasons for not using them^38, 39^. Interestingly, among differentially expressed genes of secondary follicle oocytes between pre-/mature mice, possible follicle growth regulating factors such as oxidative stress response^40, 41^, PI3K signaling^42, 43^ and lipid metabolic process^44, 45^ (enriched in mature mice) and cAMP metabolic process^46, 47^ (enriched in premature mice) were included. In the present study, relative expression levels of some of *Figla* downstream genes were significantly different between secondary follicle oocytes of pre-/mature mice, supporting that secondary follicle growth in mature mice may be regulated in part by *Figla.* However, as folliculogenesis is a highly complex process^48^ that requires appropriate set of genes to orchestrate its growth^49^, there is a limitation for discussing it with a single transcription factor.

One of the genes responsible for primary ovarian insufficiency (POI) in humans is *FIGLA*^50, 51^. Its suppression may cause POI not only through its well-known disorder in early oogenesis, but also in the subsequent follicle development failure of secondary follicles reported in the present study, which correlates with one of the typical ovarian histologies of POI patients, namely, normal-sized ovaries with partial follicular maturation^52^. Further investigations on *FIGLA* using human ovarian specimens are required to elucidate its relationship with follicle growth.

In conclusion, we herein showed for the first time that *Figla* may contribute to follicle growth in the secondary follicles of mature mice beyond its well-known function in early oogenesis through the down-regulation of stem cell signaling and estrogen signaling and up-regulation of VDR/RXR activation. These results will contribute to a better understanding of differences in folliculogenesis between premature and mature mice. A limitation of the present study is that it currently remains unclear whether the differences observed in secondary follicles between premature and mature mice are related to those between human follicles in pre-pubertal girls and adult women. Further studies using human-derived materials are needed to translate these results into clinical applications, eventually leading to the development of efficient in vitro culture systems for secondary follicles that are optimal for pre-pubertal and adult females.

## Materials and methods

### Preparation and follicle collection

ICR strain mice (CLEA Japan, Inc.) were housed in a temperature- and light-controlled environment (12L:12D) and provided with food and water *ad libitum*. Animals were maintained in accordance with the guidelines of the Science Council of Japan, and all experiments were approved by the Institutional Animal Care and Use Committee of Kyoto University (Med Kyo 15568). This study conformed to the ARRIVE guidelines^53^. Ovaries were dissected from mature (8 weeks old) and premature (10–12 days old) female mice. Secondary follicles were mechanically isolated from the ovaries using a 27G needle in L15 medium (Gibco 11415064, Thermo Fisher, US) containing 0.1% DNaseI (Sigma-Aldrich, US). Secondary follicles were selected according to the following morphological criteria^54^: two to three layers of somatic cells, the central position of the oocyte within the follicle, and a high density of somatic cells (Fig. 1a). Secondary follicles were then treated with in vitro growth (IVG) medium, which is MEMα medium (Gibco 12571-063, Thermo Fisher) supplemented with 1X insulin/transferrin/selenium solution (ITS 100X stock, Wako, Japan), 100 IU/ml penicillin & streptomycin (PenStrep, Gibco, Thermo Fisher), 5% fetal bovine serum (Sigma-Aldrich), and 0.03 IU/ml of FSH (Gonal-f 75 units, Merck Biopharma, Japan) for 1 hr.

### Preparation of siRNA reagent

All siRNA reagents were purchased from Thermo Fisher. *Figla* siRNA1# (Silencer Select Pre-Designed siRNA s77365), *Figla* siRNA2# (s77366), and negative control siRNA (Silencer Negative Control No. 1 siRNA) were used. The concentration of each reagent for injection was adjusted to 5 µM with RNase-free water.

### Microinjection and follicle culture

Secondary follicles collected from four mice were combined and randomly distributed to the negative control siRNA and *Figla* siRNA injection groups, and siRNA was microinjected into the oocytes of secondary follicles as previously reported^54^. Specifically, intact follicles were placed in M2 medium (M-7167, Sigma-Aldrich) droplets prepared in a microinjection chamber. Injection needles were prepared using a puller (Model P-97, Sutter instrument, US) with the following protocol: heat = 655, pull = 85, vel = 120, del = 110, pressure 300. An injection needle was filled with siRNA reagent and placed on a manipulator (ONM-2D, Narishige, Japan) set on an inverted microscope (IX71, Olympus, Japan). Follicles were held, the injection needle penetrated the cytoplasm of the oocyte, and siRNA reagent was injected with one shot of the FemtoJet (Eppendorf, Germany). After the microinjection, 12–13 follicles were placed on collagen-coated inserts (Transwell COL #3493, Corning, US) set in 12-well plates containing 2 ml of IVG medium in each well and incubated for 12 days (37 °C, 5% CO_2_) as previously described^55^ with slight modifications. The medium around the filter was changed every 4 days.

### Isolation of oocytes and RNA extraction

Oocytes were isolated from secondary follicles with the agitation of follicles suspended in 400 μl of MEMα medium supplemented with 0.25% collagenase (Wako) for five minutes in 1.5-ml silicon-coated tubes using a microtube mixer set in the incubator (37 °C, 5% CO_2_). Total RNA was extracted using a Nucleo Spin RNA XS (Macherey–Nagel, Germany).

### qRT-PCR

cDNA samples for qRT-PCR were prepared by applying the total RNA of oocytes to the Super Script IV VILO Master Mix (Thermo Fisher). qRT-PCR was performed using the THUNDERBIRD SYBR qPCR Mix (TOYOBO, Japan) and Applied Biosystems StepOnePlus (Thermo Fisher) (Table S3). The relative expression levels of each transcript were normalized by the 2-ΔΔCt method for endogenous GAPDH expression.

### For library preparation

cDNA products were generated and amplified by applying 1 ng of total RNA per sample to the SMART-Seq v4 Ultra Low Input RNA Kit for Sequencing (Clontech Laboratories, Inc., US), and cDNA products were quantified and evaluated using a bioanalyzer (Agilent, US) and the High Sensitivity DNA Kit (Agilent). Paired-end cDNA libraries were generated using the Nextera XT DNA Library Prep Kit (Illumina, US) by applying 0.2 ng of cDNA for each sample, and sequenced for 150 base pairs by HiSeq2500 (Illumina) for the secondary follicle oocytes of mature and premature mice and by NovaSeq6000 (Illumina) for *Figla* and control siRNA-injected secondary follicle oocytes. Raw sequencing reads were trimmed for adapter sequences and quality using Trim Galore (PMID:-, http://www.bioinformatics.babraham.ac.uk/projects/trim_galore/). The resulting sequence reads were aligned to the mouse reference genome of GRCm38/mm10 using STAR (PMID: 23104886). The mapped reads were normalized using RSEM (PMID: 21816040) to calculate expression values per gene as fragments per kilobase of transcript per million (FPKM).

### Differential gene expression, gene ontology, and pathway analyses

A differential expression analysis was performed using DESeq2 (PMID: 25516281). In comparisons of mature/premature mice, genes with FPKM > 1 for at least one sample, FDR < 0.01, and log2 fold change > 0.5 were considered as differential expressed genes (DEGs). In the comparison of si-*Figla* secondary follicle oocytes with controls, genes with FPKM > 1 for at least one sample and FDR < 0.1 were considered as DEGs. A pathway analysis was performed using Ingenuity Pathway Analysis (IPA) Software (content version: 52912811, Qiagen, Germany) and a gene ontology analysis was conducted using DAVID^56^.

### Statistical analysis

Data are presented as the mean ± *S.D.* unless otherwise noted. The two-tailed Student’s *t*-test was used to calculate *p* values. *p* < 0.05 was considered to be significant. Significance levels are symbolized by the following corresponding conditions: *p < 0.05, **p < 0.01, ***p < 0.001, and ****p < 0.0001.

## Supplementary information

Supplementary Information 1.

Supplementary Information 2.
