# Supplementary material for: Figla promotes secondary follicle growth in mature mice
Source: Sci Rep. 2021 May 10;11:9842. doi: 10.1038/s41598-021-89052-3 (PMC8110814; doi:10.1038/s41598-021-89052-3)
Supplement: Supplementary file 2 — Supplementary Information 2. [file 41598_2021_89052_MOESM2_ESM.pdf]

Supplimentary Figures (S1-S3) and Tables (S1-S3)

Figla promotes secondary follicle growth in mature mice

Asuka Okunomiya[1)], *Akihito Horie[1)], Hirohiko Tani[1)], Yukiyasu Sato[1)], Shiro Takamatsu[1)], J.B.Brown[2)], Miki Sugimoto[3)], Junzo Hamanishi[1)], Eiji Kondoh[1)], Noriomi Matsumura[4)], Masaki Mandai[1)]

1 Department of Gynecology and Obstetrics, Kyoto University Graduate School of Medicine
2 Life Science Informatics Research Unit, Department of Molecular Biosciences, Kyoto University Graduate School of Medicine
3 Laboratory of Animal Physiology and Functional Anatomy, Kyoto University Graduate School of Agriculture
4 Department of Obstetrics and Gynecology, Kindai University Faculty of Medicine
Correspondence and requests for materials should be addressed to: *
A. H. (E-mail: a_horie@kuhp.kyoto-u.ac.jp)
Department of Gynecology and Obstetrics, Kyoto University Graduate School of Medicine
54 Shogoin Kawahara-cho, Sakyo, Kyoto, 606-8507, Japan
Tel.: 81-75-751-3269, Fax: 81-75-761-3967

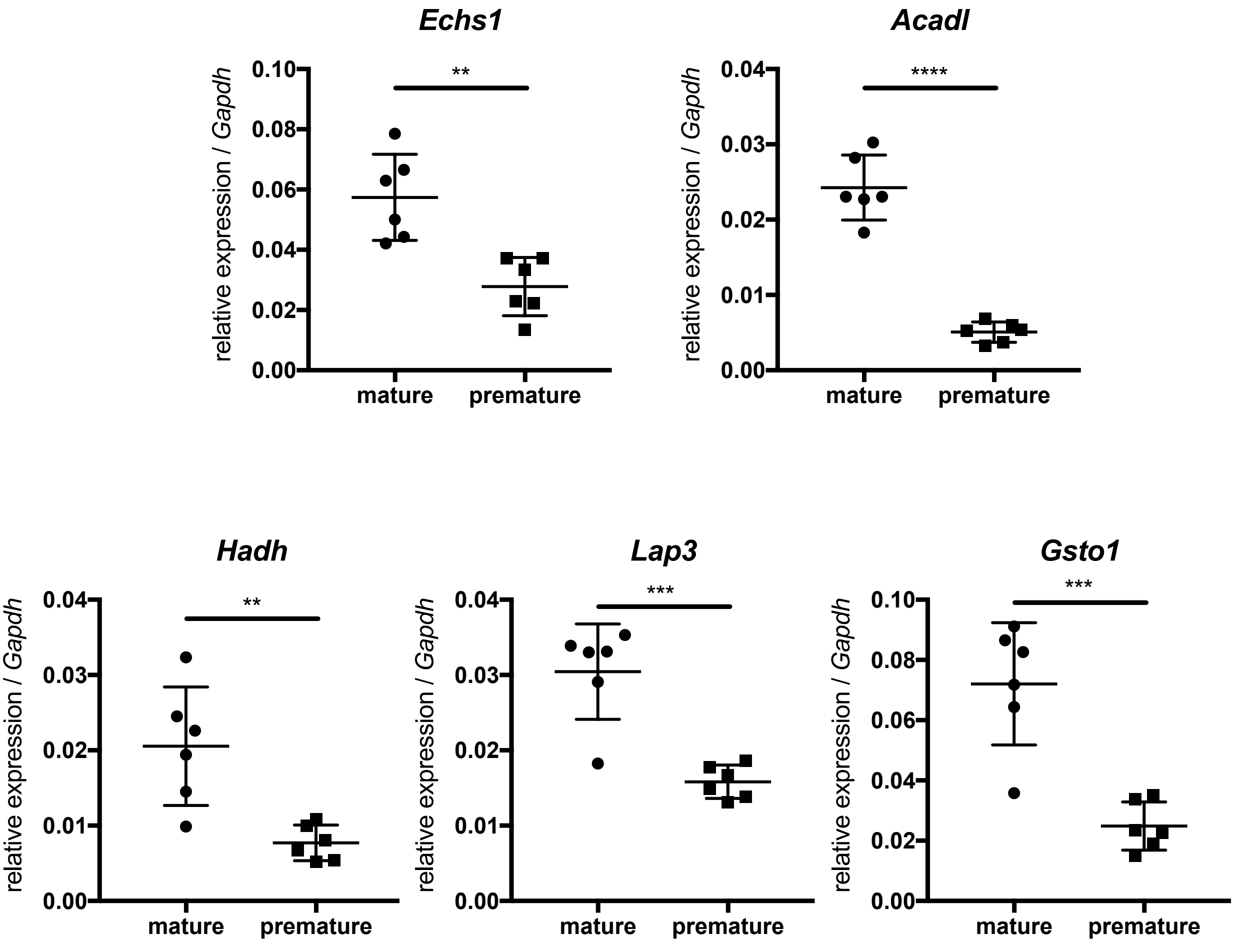

Figure S1 | Validation of oocyte RNA sequence data by qRT-PCR

Table S1-1 Gene ontology analysis of differentially expressed genes up-regulated in mature mice

GO term	Description	p-value	Gene count
GO:0016310	phosphorylation	4.00E-05	41
GO:0008152	metabolic process	1.92E-04	32
GO:0006749	glutathione metabolic process	1.95E-04	9
GO:0009142	nucleoside triphosphate biosynthetic process	4.86E-04	5
GO:0055114	oxidation-reduction process	6.33E-04	40
GO:0051289	protein homotetramerization	7.61E-04	10
GO:0045944	positive regulation of transcription from RNA polymerase II promoter	8.80E-04	53
GO:0001649	osteoblast differentiation	0.00137	12
GO:0006629	lipid metabolic process	0.00159	29
GO:0071277	cellular response to calcium ions	0.00179	8
GO:0048167	regulation of synaptic plasticity	0.00251	7
GO:0006228	UTP biosynthetic process	0.00265	4
GO:0030036	actin cytoskeleton organization	0.00284	13
GO:0045746	negative regulation of the Notch signaling pathway	0.00333	6
GO:0035690	cellular response to drugs	0.00342	9

Table S1-2 Gene ontology analysis of differentially expressed genes up-regulated in premature mice

GO term	Description	p-value	Gene count
GO:0046069	cGMP catabolic process	6.52E-04	4
GO:0050873	brown fat cell differentiation	0.00109	6
GO:0001525	angiogenesis	0.001567	15
GO:0097113	AMPA glutamate receptor clustering	0.00521	3
GO:0006198	cAMP catabolic process	0.00567	4
GO:0055085	transmembrane transport	0.00569	18
GO:0035556	intracellular signal transduction	0.00630	19
GO:0035092	sperm chromatin condensation	0.00769	3
GO:0007155	cell adhesion	0.0104	21
GO:0006303	double-strand break repair via non-homologous end joining	0.0124	4
GO:0045494	photoreceptor cell maintenance	0.0139	5
GO:1902042	negative regulation of the extrinsic apoptotic signaling pathway via death domain receptors	0.0200	4
GO:0015909	long-chain fatty acid transport	0.0217	3
GO:0071340	skeletal muscle acetylcholine-gated channel clustering	0.0217	3
GO:0007411	axon guidance	0.0241	9

a **b**

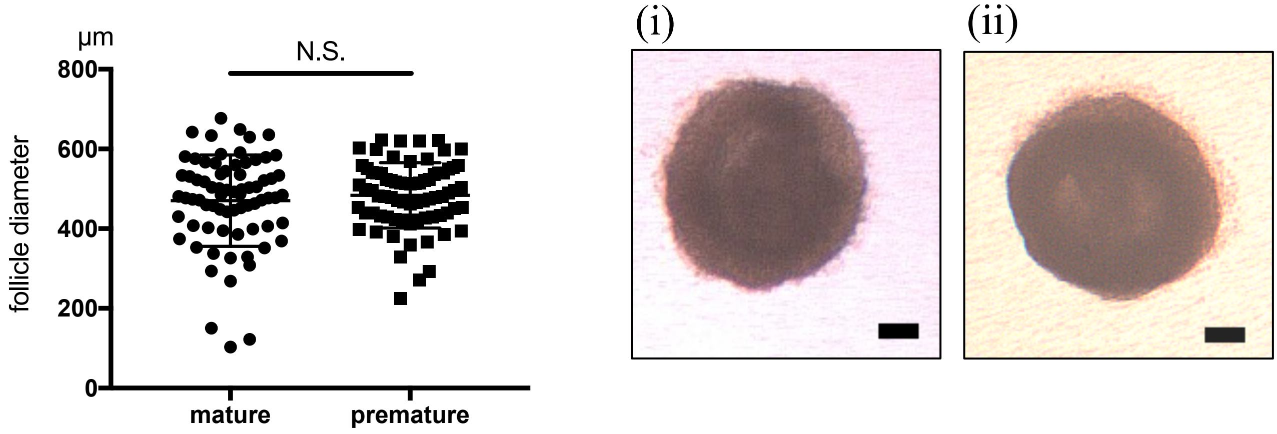

Figure S2 | Diameter of follicles after 12 days of *in vitro* culture

(a) The diameter of follicles after 12 days of *in vitro* culture was not significantly different between mature and premature mice. Follicle diameters were 470.4 ± 114.8 µm (n=73) in mature mice and 483.8 ± 81.99 (n=75) in premature mice. (b) Representative pictures of follicles after *in vitro* culture. (i) Mature mouse, (ii) Premature mouse. Scale bars, 100 µm.

Table S2-1 Top 15 canonical pathways of differentially expressed genes between newborn ovaries of *Figla*_null mice and control mice

Ingenuity Canonical Pathways	p-value	[a]Ratio	[b]z-score	[c]Genes
Molecular Mechanisms of Cancer	1E-11	0.292	N/A	Abl1,**Adcy10**,Adcy2,Adcy3,**Arhgef16**,Arhgef17,Arhgef19,Arhgef5,Arhgef9,**Atr**,**Aurka**,**Bax**,Bcl2l11,**Bid**,Birc2,Bmp1,**Bmp15**,Bmp2,**Camk2b**,Casp9,Ccnd1,Ccnd2,**Cdc25a**,**Cdc42**,**Cdh1**,**Cdk1**,Cdk14,Cdk4,Cdk6,**Cdk8**,**Cdkn2a**,**Chek1**,**Crebbp**,Ctnna1,Ctnnb1,Ctnnd1,**Diablo**,**E2f1**,**E2f5**,**Fancd2**,Fzd1,Fzd10,Fzd2,Fzd4,**Fzd5**,Fzd6,Fzd7,Fzd8,**Gna11**,Gnai2,Gnai3,Gnao1,Gnas,Hat1,Hhat,**Hras**,Irs1,Itga5,**Jak2**,**Lamtor3**,Lef1,Lrp1,**Map2k1**,**Map3k5**,Mapk10,**Mapk8**,**Mdm2**,**Pak2**,**Pak4**,Pik3c2a,Pik3c2b,**Pik3cb**,**Pik3cd**,Pik3r1,Pik3r3,**Pik3r4**,Pik3r6,Plcb1,Plcb3,**Prkag2**,Prkcb,Prkci,Prkdc,**Psen2**,**Ptch1**,**Ralbp1**,Rap2b,**Rasd2**,Rasgrf1,**Rb1**,Rhob,Rhoc,Rhoj,Rhoq,Rnd3,Shc1,**Smad3**,Smad5,Smad6,Smad7,Smad9,Smo,**Stk36**,Sufu,Syngap1,Tgfb2,Tgfbr2,Wnt2b,Wnt4,Wnt5a,Wnt5b,**Wnt7a,Zbtb17**
Axonal Guidance Signaling	2.00E-11	0.275	N/A	Abl1,**Ablim1**,**Ablim2**,Ablim3,**Actr3**,**Adam12**,Adam15,Adam19,Adamts10,Adamts12,Adamts16,**Adamts18**,Adamts19,Adamts2,Adamts3,Adamts7,**Afg3l2**,Arpc1b,**Arpc3**,**Arpc5l**,Baiap2,Bmp1,**Bmp15**,Bmp2,**Cfl1**,**Cfl2**,Cxcl12,Dpysl2,Efna5,Efnb1,Efnb3,**Eif4e**,Epha7,Ephb2,Ephb4,Fes,Fzd1,Fzd10,Fzd2,Fzd4,**Fzd5**,Fzd6,Fzd7,Fzd8,Gli2,Gli3,**Glis1**,Glis2,**Gna11**,Gnai2,Gnai3,Gnao1,Gnas,Gng2,Herc2,**Hras**,Igf1,Itga5,**Kalrn**,Kif7,**Limk1**,**Map2k1**,Mme,Mmp14,Mmp15,Mmp16,Mmp2,Mmp28,Myl9,**Nck2**,Nfatc1,**Nfatc2**,Notum,Nrp1,Nrp2,Ntn4,Ntng1,**Pak2**,**Pak4**,Pappa,**Pik3c2a**,**Pik3c2b**,**Pik3cb**,**Pik3cd**,Pik3r1,Pik3r3,**Pik3r4**,Pik3r6,Plcb1,Plcb3,**Plcg2**,Plxna3,Plxnd1,**Ppp3cb**,**Prkag2**,Prkcb,Prkci,**Ptch1**,Pxn,Rap2b,**Rasd2**,Robo2,**Rock1**,Rtn4,Sema3a,Sema3g,Sema5a,Sema6a,Sema6c,Sema6d,Sema7a,Shc1,Slit2,Slit3,Smo,Srgap1,Srgap3,**Stk36**,Sufu,**Tuba1c**,**Tubb2b**,**Tubb4b**,Unc5b,Unc5d,Vegfb,**Wasl**,Wnt2b,Wnt4,Wnt5a,Wnt5b,**Wnt7a**
p53 Signaling	7.76E-09	0.398	-0.962	**Atr**,**Bax**,**Birc5**,Ccnd1,Ccnd2,**Ccnk**,Cdk4,**Cdkn2a**,**Chek1**,Ctnnb1,**E2f1**,Gadd45b,Gadd45g,Hdac9,**Mapk8**,**Mdm2**,**Mdm4**,**Pcna**,**Perp**,**Pik3c2a**,**Pik3c2b**,**Pik3cb**,**Pik3cd**,Pik3r1,Pik3r3,**Pik3r4**,Pik3r6,**Ppp1r13b**,Prkdc,**Rb1**,**Rrm2b**,Snai2,**St13**,Thbs1,**Tigar**,**Trim29**
Role of NANOG in Mammalian Embryonic Stem Cell Pluripotency	1.26E-08	0.37	0.447	Bmp1,**Bmp15**,Bmp2,Ctnnb1,Fzd1,Fzd10,Fzd2,Fzd4,**Fzd5**,Fzd6,Fzd7,Fzd8,Gata4,Gata6,**Hras**,Il6st,**Jak2**,Lifr,**Map2k1**,**Pik3c2a**,**Pik3c2b**,**Pik3cb**,**Pik3cd**,Pik3r1,Pik3r3,**Pik3r4**,Pik3r6,**Pou5f1**,Rap2b,**Rasd2**,**Rif1**,Shc1,Smad5,Smad9,Smo,Sox2,**Stat3**,Tcf7l1,Wnt2b,Wnt4,Wnt5a,Wnt5b,**Wnt7a**
Basal Cell Carcinoma Signaling	2.95E-08	0.431	-2.353	Apc2,Bmp1,**Bmp15**,Bmp2,Ctnnb1,**Dvl3**,Fzd1,Fzd10,Fzd2,Fzd4,**Fzd5**,Fzd6,Fzd7,Fzd8,Gli2,Gli3,**Glis1**,Glis2,Kif7Llef1,**Ptch1**,Smo,**stk36**,Sufu,Tcf7l1,Tcf7l2,Wnt2b,Wnt4,Wnt5a,Wnt5b,**Wnt7a**
Hereditary Breast Cancer Signaling	4.57E-08	0.343	N/A	**Atr**,**Bard1**,**Ccnb1**,Ccnd1,**Cdk1**,Cdk4,Cdk6,**Chek1**,**Crebbp**,**E2f1**,**Faap100**,**Fancc**,**Fancd2**,Gadd45b,Gadd45g,**Hdac11**,Hdac2,Hdac5,Hdac7,Hdac9,**Hras**,**Msh6**,**Phf10**,**Pik3c2a**,**Pik3c2b**,**Pik3cb**,**Pik3cd**,Pik3r1,Pik3r3,**Pik3r4**,Pik3r6,Polr2a,**Polr2d**,**Polr2e**,**Polr2g**,**Polr2j**,**Polr2k**,**Rad50**,**Rad51**,Rap2b,**Rasd2**,**Rb1**,**Rfc1**,**Rfc4**,**Rpa1**,**Smarcb1**,Smarcd3,**Wee1**

Ingenuity Canonical Pathways	p-value	[a]Ratio	[b]z-score	[c]Genes
Germ Cell-Sertoli Cell Junction Signaling	6.17E-08	0.322	N/A	A2m,Acta2,**Actn3**,**Afdn**,**Cdc42**,**Cdh1**,**Cfl1**,**Cfl2**,**Clint1**,Ctnna1,Ctnnb1,Ctnnd1,**Epn2**,Gsn,**Hras**,Jup,Lamc3,**Limk1**,**Map2k1**,**Map3k11**,Map3k12,**Map3k4**,**Map3k5**,**Map3k6**,**Map3k9**,Mapk10,**Mapk8**,**Pak2**,**Pak4**,**Pdpk1**,**Pik3c2a**,**Pik3c2b**,**Pik3cb**,**Pik3cd**,Pik3r1,Pik3r3,**Pik3r4**,Pik3r6,**pls1**,Pxn,Rap2b,**Rasd2**,Rhob,Rhoc,Rhoj,Rhoq,Rnd3,Tgfb2,Tgfbr2,Tnfrsf1a,**Tuba1c**,**Tubb2b**,**Tubb4b**
Hepatic Fibrosis / Hepatic Stellate Cell Activation	2.29E-07	0.306	N/A	A2m,Acta2,Bambi,**Bax**,Col12a1,Col13a1,Col15a1,cCl16a1,**Col18a1**,Col1a1,Col1a2,Col22a1,Col23a1,**Col24a1**,Col27a1,Col3a1,Col5a1,Col5a2,Col5a3,Col6a2,Col6a3,Col7a1,Col8a2,Col9a2,**Col9a3**,Edn1,Ednra,Fgfr2,Flt4,Fn1,Ifnar2,Igf1,Igf2,Igfbp4,Igfbp5,Il10ra,**Il1r1**,Il1rl2,Kdr,Klf6,Mmp2,Myh10,Myh11,Myh3,Myl9,Pdgfra,Pdgfrb,**Smad3**,Smad7,Tgfb2,Tgfbr2,Timp2,Tnfrsf1a,Tnfrsf1b,Vegfb
Glioblastoma Multiforme Signaling	2.88E-07	0.315	-3.244	Ccnd1,**Cdc42**,Cdk4,Cdk6,**Cdkn2a**,Ctnnb1,**E2f1**,**E2f5**,Fzd1,Fzd10,Fzd2,Fzd4,**Fzd5**,Fzd6,Fzd7,Fzd8,**Hras**,Igf1,Igf2,**Itpr1**,Lef1,**Map2k1**,**Mdm2**,Notum,Pdgfra,Pdgfrb,**Pik3c2a**,**Pik3c2b**,**Pik3cb**,**Pik3cd**,Pik3r1,Pik3r3,**Pik3r4**,Pik3r6,Plcb1,Plcb3,**Plcg2**,Rap2b,**Rasd2**,**Rb1**,Rhob,Rhoc,Rhoj,Rhoq,Rnd3,Shc1,Smo,Wnt2b,Wnt4,Wnt5a,Wnt5b,**Wnt7a**
Human Embryonic Stem Cell Pluripotency	3.02E-07	0.333	N/A	Acvr1,Bmp1,**Bmp15**,Bmp2,Ctnnb1,Fgfr2,Fzd1,Fzd10,Fzd2,Fzd4,**Fzd5**,Fzd6,Fzd7,Fzd8,Gnas,Lef1,Pdgfra,Pdgfrb,**Pdpk1**,**Pik3c2a**,**Pik3c2b**,**Pik3cb**,**Pik3cd**,Pik3r1,Pik3r3,**Pik3r4**,Pik3r6,**Pou5f1**,S1pr1,S1pr3,**Smad3**,Smad5,Smad6,Smad7,Smo,Sox2,Tcf7l1,Tcf7l2,Tgfb2,Tgfbr2,Wnt2b,Wnt4,Wnt5a,Wnt5b,**Wnt7a**
Ovarian Cancer Signaling	7.59E-07	0.324	-0.655	Abl1,Ccnd1,Cdk4,**Cdkn2a**,Ctnnb1,**E2f1**,Edn1,Ednra,Fzd1,Fzd10,Fzd2,Fzd4,**Fzd5**,Fzd6,Fzd7,Fzd8,Gja1,**Hras**,Lef1,**Map2k1**,Mmp2,**Msh6**,**Pik3c2a**,**Pik3c2b**,**Pik3cb**,**Pik3cd**,Pik3r1,Pik3r3,**Pik3r4**,Pik3r6,**Prkag2**,**Rad51**,Rap2b,**Rasd2**,**Rb1**,Smo,Tcf7l1,Tcf7l2,Vegfb,Wnt2b,Wnt4,Wnt5a,Wnt5b,**Wnt7a**
GP6 Signaling Pathway	1.05E-06	0.336	-2.53	Col12a1,Col13a1,Col15a1,Col16a1,**Col18a1**,Col1a1,Col1a2,Col22a1,Col23a1,**Col24a1**,Col27a1,Col3a1,Col5a1,Col5a2,Col5a3,Col6a1,Col6a2,Col6a3,Col7a1,Col8a2,Col9a2,**Col9a3**,**Itpr1**,Lama3,Lamb2,Lamc3,**Nox1**,**Pdpk1**,**Pik3c2a**,**Pik3c2b**,**Pik3cb**,**Pik3cd**,Pik3r1,Pik3r3,**Pik3r4**,Pik3r6,**Plcg2**,Prkcb,Prkci,Tln1
Mouse Embryonic Stem Cell Pluripotency	1.26E-06	0.35	-1.183	**Crebbp**,Ctnnb1,**Dvl3**,Fzd1,Fzd10,Fzd2,Fzd4,**Fzd5**,Fzd6,Fzd7,Fzd8,**Hras**,**Id1**,Il6st,**Jak2**,Lef1,Lifr,**Map2k1**,**Pik3c2a**,**Pik3c2b**,**Pik3cb**,**Pik3cd**,Pik3r1,Pik3r3,**Pik3r4**,Pik3r6,**Pou5f1**,Rap2b,**Rasd2**,Smad5,Smad9,Smo,Sox2,**Stat3**,Tcf7l1,Tcf7l2
Hepatic Fibrosis Signaling Pathway	1.29E-06	0.253	-3.362	Acta2,Acvr1,Bambi,Cacna1a,Ccnd1,**Cdc42**,Cnr1,**Col18a1**,Col1a1,Col1a2,Col3a1,Col5a3,**Crebbp**,Ctnnb1,**Dvl3**,Edn1,Ednra,Flt4,Foxo4,Fzd1,Fzd10,Fzd2,Fzd4,**Fzd5**,Fzd6,Fzd7,Fzd8,Gli2,Gli3,**Glis1**,Glis2,Gnai2,Gnai3,**Hras**,**Ikbkb**,**Il1r1**,Il1rl2,Irs2,Itga5,**Jak2**,Kdr,Klf9,Lef1,**Map2k1**,Mapk10,**Mapk8**,Myl9,Mylk,**Nox1**,Pdgfra,Pdgfrb,**Pdk1**,**Pik3c2a**,**Pik3c2b**,**Pik3cb**,**Pik3cd**,Pik3r1,Pik3r3,**Pik3r4**,Pik3r6,**Plcg2**,**Prkag2**,Prkcb,Prkci,**Ptch1**,Rap2b,**Rasd2**,Rhob,Rhoc,Rhoj,Rhoq,Rnd3,**Rock1**,**Sdhb**,**Smad3**,Smad7,Smo,**Stat3**,Tcf7l1,Tcf7l2,Tgfb2,Tgfbr2,Tnfrsf1a,Tnfrsf1b,**Ttn**,Vegfb,Wnt2b,Wnt4,Wnt5a,Wnt5b,**Wnt7a**
Regulation Of The Epithelial Mesenchymal Transition In The Development Pathway	1.82E-06	0.369	-2.268	Axin2,**Cdh1**,ctnnb1,**Dvl3**,Fzd1,Fzd10,Fzd2,Fzd4,**Fzd5**,Fzd6,Fzd7,Fzd8,Gli2,Gli3,**Glis1**,Glis2,**Jag1**,**Jag2**,Lef1,**Psen2**,**Ptch1**,Smo,Snai2,Sufu,Tcf7l1,Tcf7l2,Wnt2b,Wnt4,Wnt5a,Wnt5b,**Wnt7a**

[a]**Ratio** of listed genes found in each pathway over the total number of genes in that pathway

[b]**z-score** positive when pathways are down-regulated in *Figla*_null mice (= pathways are expected to be up-regulated by *Figla*), while negative when pathways are up-regulated in *Figla*_null mice (= pathways are expected to be down-regulated by *Figla*)

[c]**genes** in bold are down-regulated in *Figla*_null mice (= genes expected to be up-regulated by *Figla*), while genes in normal font are up-regulated in *Figla*_null mice (= genes expected to be down-regulated by *Figla*)

Table S2-2 Gene ontology analysis of differentially expressed genes down-regulated in *Figla*_null mice

GO term	Description	p-value	Gene count
GO:0007049	cell cycle	4.68E-25	148
GO:0051301	cell division	8.53E-24	106
GO:0007067	mitotic nuclear division	7.00E-23	87
GO:0034587	piRNA metabolic process	1.62E-09	17
GO:0006974	cellular response to a DNA damage stimulus	7.64E-09	81
GO:0007059	chromosome segregation	1.73E-08	29
GO:0051321	meiotic cell cycle	8.36E-08	29
GO:0043046	DNA methylation involved in gamete generation	1.48E-07	12
GO:0006260	DNA replication	2.56E-07	33
GO:0006281	DNA repair	3.54E-07	62
GO:0051028	mRNA transport	8.76E-07	26
GO:0006810	transport	1.05E-06	242
GO:0031047	gene silencing by RNA	1.75E-06	19
GO:0006417	regulation of translation	1.80E-06	31
GO:0042254	ribosome biogenesis	3.97E-06	25

Table S2-3 Gene ontology analysis of differentially expressed genes up-regulated in *Figla*_null mice

GO term	Description	p-value	Gene count
GO:0007155	cell adhesion	1.17E-29	111
GO:0007275	multicellular organism development	1.53E-24	164
GO:0007507	heart development	5.34E-17	61
GO:0007411	axon guidance	2.45E-16	44
GO:0001525	angiogenesis	1.35E-15	56
GO:0030154	cell differentiation	7.81E-14	113
GO:0030336	negative regulation of cell migration	2.84E-12	32
GO:0007179	transforming growth factor beta receptor signaling pathway	1.01E-10	25
GO:0000122	negative regulation of transcription from RNA polymerase II promoter	1.41E-10	99
GO:0016477	cell migration	2.20E-10	41
GO:0008285	negative regulation of cell proliferation	3.28E-10	63
GO:0030335	positive regulation of cell migration	4.22E-10	42
GO:0016055	Wnt signaling pathway	5.64E-10	43
GO:0043627	response to estrogen	6.43E-10	24
GO:0045893	positive regulation of transcription, DNA-templated	7.13E-10	82

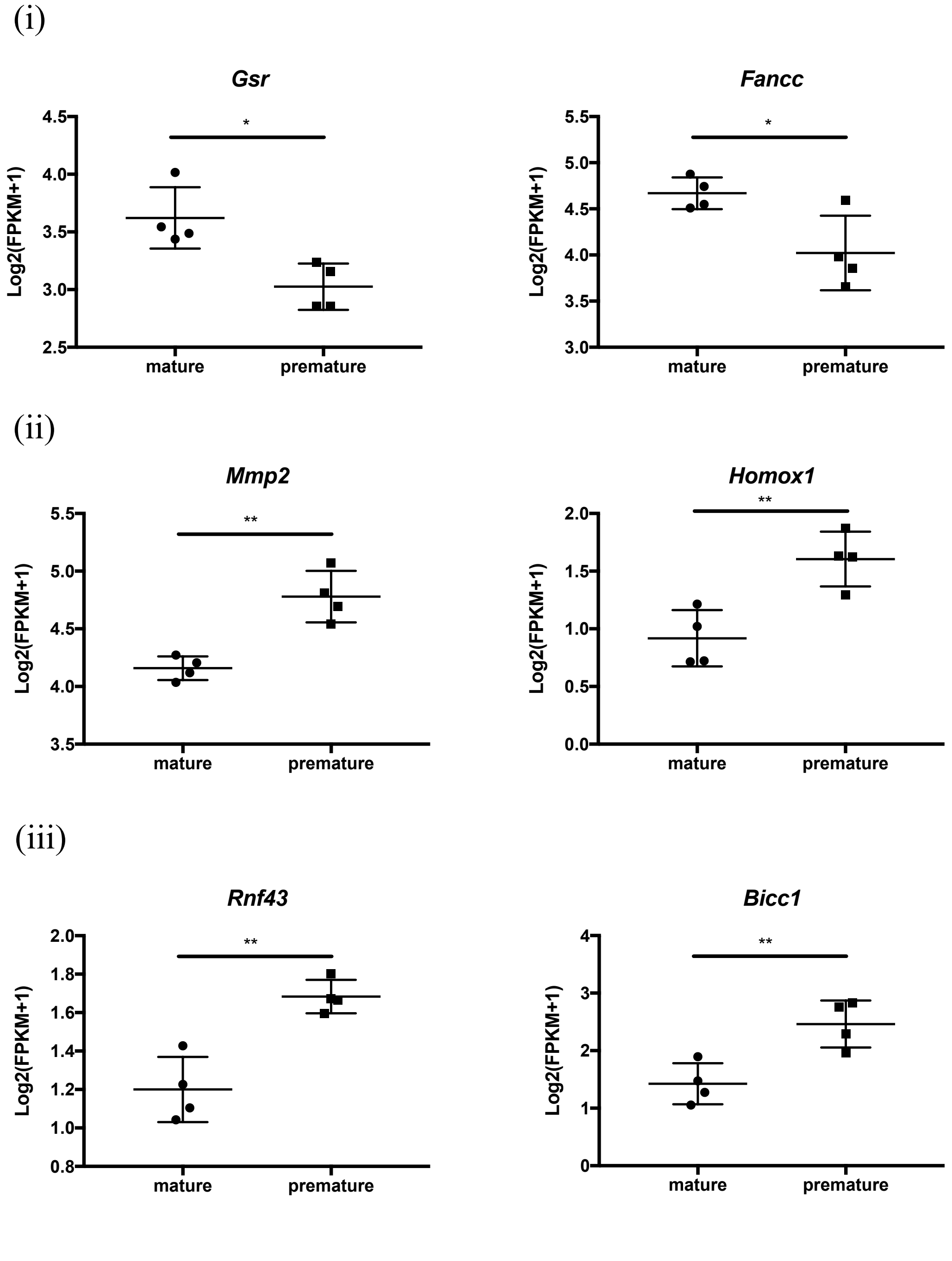

Figure S3 | Relative expression levels of *Figla* downstream genes in secondary follicle oocytes of mature and premature mice

Among *Figla* downstream genes, relative expression levels of (i)*Gsr* and *Fancc* (genes related to oxidative stress response)*,* which were expected to be up-regulated by *Figla*, were significantly higher in mature mice than premature mice, whereas those of (ii)*Mmp2* and *Hmox1* (genes related to estrogen signaling) and (iii)*Rnf43* and *Bicc1* (genes related to stem cell signaling), which were expected to be down-regulated by *Figla*, were significantly lower in mature mice than premature mice.

Gene	Forward primer 5'-3'	Reverse primer 5'-3'
Gapdh	AACTCCCACTCTTCCACCTTCG	CCCTGTTGCTGTAGCCGTATTC
Figla	AGATACTCGGCTGTGTTCTGGAAG	GCTGGTAGGTTGGGTAGCATTTC
Echs1	ACCTTTGCCACCGATGACC	GGACAGGCTGAGCTTTCCAG
Acadl	TGGTGGAAAACGGAATGAAAGG	AATAGTTCTGCTGTGTCCCTGAGC
Hadh	GAGGCGATGCGTCTAAGGAA	TCCATTTCATGCCACCCGTC
Lap3	TGAGGGCTGATATGGGAGGA	CAAAGAGGGGCCAAACCAATG
Gsto1	TAATTTGACCTTCTGGCCTA	GAAAGTATGGGGAAATCACA

Table S3 | Primers for qRT-PCR
